# A feasible diagnostic approach for the translocation carrier from the indication of products of conception

**DOI:** 10.1186/s13039-018-0362-8

**Published:** 2018-01-30

**Authors:** Ye-Qing Qian, Xiao-Ying Fu, Xiao-Qing Wang, Yu-Qin Luo, Min Chen, Kai Yan, Yan-Mei Yang, Bei Liu, Li-Ya Wang, Ying-Zhi Huang, Hong-Ge Li, Hang-Yi Pan, Fan Jin, Min-Yue Dong

**Affiliations:** 1 Women’s Hospital, School of Medicine, Zhejiang University, 1, Xueshi Road, Hangzhou, Zhejiang 310006 People’s Republic of China; 2 Key Laboratory of Reproductive Genetics (Zhejiang University), Ministry of Education, 1, Xueshi Road, Hangzhou, Zhejiang 310006 People’s Republic of China; 3 Key Laboratory of Women’s Reproductive Health of Zhejiang Province, 1, Xueshi Road, Hangzhou, Zhejiang 310006 People’s Republic of China

**Keywords:** Chromosome microarray analysis, Balanced translocation, Fluorescence in situ hybridization, Products of conception

## Abstract

**Background:**

Chromosome translocations are rare but frequently associated with infertility. The objective of this study is to investigate the feasibility of using chromosomal microarray analysis (CMA) on products of conception (POC) samples as an indicator of parental balanced translocation. From January 2011 to December 2016, CMA using Affymetrix Cytoscan™750K array was performed on 1294 POC samples in our hospital. Karyotyping and fluorescence in situ hybridization (FISH) using parental blood samples were performed to validate the origin of subchromosomal copy number variations (CNVs).

**Results:**

In the 1294 cases of POCs, we detected CNVs of terminal duplication and deletion that imply unbalanced translocation derivatives in 16 cases, and accurate diagnosis with the parental study was made in all the cases by karyotyping and/or FISH. In 10/16 (62.5%) of these cases, CNVs were inherited from one carrier parent of balanced translocation (Cases 1 to 10), while 6/16 (37.5%) cases occurred de novo (Cases 11 to 16).

**Conclusion:**

This study clearly illustrated the importance of the utilization of CMA on POC, followed by parental karyotyping and FISH to better characterize CNVs. This approach is especially useful for couples in whom one partner carries a cryptic/submicroscopic balanced translocation but has an apparently normal karyotype.

**Electronic supplementary material:**

The online version of this article (10.1186/s13039-018-0362-8) contains supplementary material, which is available to authorized users.

## Background

Chromosomal microarray analysis (CMA) is a technique of identifying major chromosomal aneuploidy and submicroscopic abnormalities that are too small to be detected by conventional karyotyping, thus providing information at the submicroscopic level throughout the whole human genome. In addition, SNP-based CMA can also identify polyploidy, whole-genome homozygosity, uniparental disomy, parental relatedness and maternal cell contamination, thus maximizing sensitivity and decreasing false-negative results [1–3].

CMA is also a powerful tool to detect small chromosome duplications and/or deletions in kilobase range known as copy number variations (CNVs) that are not detectable by karyotyping. This advantage has already enabled CMA showing its value in post- and prenatal diagnostics. Miller et al. had recommended CMA as a first-tier clinical diagnostic test for individuals with developmental disabilities or congenital anomalies [4]. Moreover, Wapner et al. concluded that CMA could detect additional clinically relevant genomic disorders as compared with karyotyping in the prenatal diagnosis [5]. Likewise, Reddy and his colleagues reported the valuable application of CMA in the analyses of stillbirths, that is, CMA provided a relative increase in the diagnosis of chromosomal abnormalities with nonviable tissues as compared with karyotyping, which required cell culture processes [6–8].

Translocation is one type of chromosomal abnormalities that occur when chromosomes break and the fragments re-join other chromosomes. If this event happened between two nonhomologous chromosomes, derivative chromosomes would form. Reciprocal translocation, the most common type of translocation, can further be classified into balanced and unbalanced translocation according to whether the genetic materials change with gain/loss (unbalanced) or not (balanced). Unbalanced translocation is always presented with partial trisomy for one chromosome and partial monosomy for the other chromosome [9].

In order to investigate the feasibility of using CMA on POC samples as an indicator of parental balanced translocation for couples experienced at least one spontaneous miscarriage and/or fetal abnormalities, we conducted a retrospective study on 1294 POC samples collected during the past 6 years in our hospital. Among the 1294 cases, we identified 16 cases of samples with partial trisomy and monosomy in different chromosomes. We showed that discovery of terminal deletion and duplication of POC samples by CMA may indicate an unbalanced translocation (62.5%, 10 in 16 cases). CMA on POC samples is of great importance when one of the couple is balanced translocation carrier but could not be distinguished by conventional karyotype.

## Methods

### Participants and samples

From January 2011 to December 2016, a total of 1294 POC samples were investigated. CMA analysis was performed on all POC samples including chorionic villi samples (CVSs) from women who had spontaneous miscarriages or tissue (muscle) of the fetus with congenital anomalies. Each sample was rinsed in normal saline solution three times. Then 10 mg of each tissue were submitted to genomic DNA extraction. Fifteen CVSs and one tissue samples were found having partial trisomy for one chromosome and partial monosomy for the other chromosome. Parental studies using karyotyping and/or FISH technologies were performed on all the cases. The information of these cases was summarized in Table 1, and Case 1 and 2 were described in detail as follows:

**Table 1:** Origin analysis of POC samples with segmental CNVs by parental karyotyping or FISH

Case	Array Results for POC samples	MLPA results	Maternal karyotype	Paternal karyotype	FISH	Parents origin	Medical history and follow-up
Parental inherited cases							
1	arr 6q27(165,051,708–170,914,297)× 1(5.8 Mb), 17p13.3p12(525–11,208,838)× 3 (11.2 Mb)	Del 6q (TBP-2) Dup 17p (RPH3AL-2)	46,XX	46,XY	Yes	Maternal	Detailed in this article
2	arr 2q37.1q37.3(231,919,970–242,782,258)× 3(10.8 Mb), 17p13.3p12(525–13,548,932)× 1 (13.5 Mb)	Dup 2q (ATG4B-7) Del 17p (RPH3AL-2)	46,XX	46,XY	Yes	Maternal	Detailed in this article
3	arr 13q31.1q34(80,538,198–115,019,701)×1 (34 Mb), 16q12.1q24.3(50,917,739–90,147,202)× 3 (39 Mb)	Del 13q (CDC16–8) Dup 16q (GAS8–11)	46,XX	46,XY	Yes	Paternal	33 years old, G2P0 (inevitable abortion twice), turned to PGD (not performed yet)
4	arr 4q32.2q35.2(164,135,613–190,957,460)× 1 (26.8 Mb), 6q27(167,838,939–170,914,297)× 3 (3 Mb)	Del 4q (FRG1–1) Dup 6q (TBP-2)	46,XX,t(4;6) (q31.3;q25)	46,XY	No	Maternal	30 years old, G2P0 (inevitable abortion twice), turned to PGD (4 embryos biopsied, 3 normal embryos), give birth to healthy twin boys on Feb 2016
5	arr 4p16.3(68,345–2,749,375)× 1 (2.6 Mb), 9p24.3p13.1(208,454–38,787,479)× 3 (38.5 Mb)	Del 4p (PIGG-8) Dup 9p (DOCK8–24)	46,XX,t(4;9) (p16.3;p13)	46,XY	No	Maternal	30 years old, G1P0 (inevitable abortion once), recently have a healthy boy (no karyotype information)
6	arr 8p23.3p22(158,048–14,429,347)×1 (14.2 Mb), 12p13.33p12.2(173,786–21,214,526)× 3 (21 Mb)	Del 8p (FBXO25–8) Dup 12p (KDM5A-23)	46,XX	46,XY,t(8;12) (p22;p12)	No	Paternal	31 years old, G2P0 (inevitable abortion twice)
7	arr 3q29(195,718,751–197,851,444)×3 (2.1 Mb), 18q21.1q23(47,069,039–78,013,728)× 1 (30.9 Mb)	Dup 3q (KIAA0226–2) Del 18q (CTDP1–9)	46,XX,t(3;18) (q29;q21.1)	46,XY	No	Maternal	32 years old, G2P0 (inevitable abortion twice), turn to PGD (8 embryos biopsied, no normal embryos)
8	arr 1p36.33(849,466–1,130,311)×1 (281 kb), 7q31.33q36.3(126,696,825–159,119,707)× 3 (32.4 Mb)	Del 1p (TNFRSF18–4) Dup 7q (VIPR2–2)	46,XX	46,XY,t(1;7) (p36.3;q32)	No	Paternal	34 years old, G2P0 (inevitable abortion twice)
9	arr 10q25.3q26.3(18,616,597–135,434,149)×3 (16 Mb), 11p15.5(196,990–46,712,144)× 1 (46 Mb)	Dup 10q (ECHS1–8) Del 11p (BET1L-3)	46,XX,t(10;11) (q26;p13)	46,XY	No	Maternal	37 years old, G1P0 (inevitable abortion once), turned to PGD (2 embryos biopsied, no normal embryos)
10	arr 8p23.3p12(158,048–30,640,081)×1 (30.5 Mb), 16q23.2q24.3(81,433,414–90,155,062)× 3 (8.7 Mb)	Del 8p (FBXO25–8) Dup 16q (GAS8–11)	46,XX	46,XY,t(8;16) (p21;q24)	No	Paternal	31 years old, G2P0 (inevitable abortion twice)
De novo cases							
11	arr 7p22.3p21.3(43,376–13,484,133)×3 (13.4 Mb), 21q22.3(46,873,868–48,093,361)× 1 (1.2 Mb)	Dup 7p (SUN1–5) Del 21q (S100B-2)	46,XX	46,XY	Yes	De novo	25 years old, G1P0 (inevitable abortion once)
12	arr 15q26.1q26.3(92,007,796–102,429,040)×1 (10.4 Mb), 20p13p12.2(61,661–10,622,608)× 3 (10.5 Mb)	Del 15q (TM2D3–3) Dup 20p (ZCCHC3–1)	46,XX	46,XY	Yes	De novo	30 years old, G3P0 (inevitable abortion three times)
13	arr 2q37.3(240,574,853–242,783,384)×1 (2 Mb), 14q32.31q32.33(102,767,052–107,152,784)× 3 (4 Mb)	Del 2q (ATG4B-7) Dup 14q (MTA1–7)	46,XX	46,XY	Yes	De novo	30 years old, G2P0 (inevitable abortion twice)
14	arr 3p26.3p25.3(61,891–10,459,934)×1 (10.3 Mb), 9p24.3p21.3(208,454–21,834,597)× 3 (21.6 Mb)	Del 3p (CHL1–3) Dup 9p (DOCK8–24)	46,XX	46,XY	Yes	De novo	38 years old, G1P0 (inevitable abortion once), recently have a healthy girl (no karyotype information)
15	arr 3p26.3p26.1(61,891–7,990,630)× 1 (7.9 Mb), 7q34q36.3(139,958,543–159,119,707)×2~ 3 (19.1 Mb)	Del 3p (CHL1–3)	46,XX	46,XY,inv.(1) (p13;q21)	No	De novo	34 years old, G1P0 (inevitable abortion once), recently have a healthy boy (no karyotype information)
16	arr 3p26.3p21.2(61,891–52,187,541)×3 (52 Mb), 11q24.1q25(121,869,825–134,937,416)× 1 (13 Mb)	Dup 3p (CHL1–3) Del 11q (IGSF9B-20)	46,XX	46,XY	No	De novo	32 years old, G2P1 (inevitable abortion once), recently have a healthy boy (no karyotype information)

#### Case 1

Thirty years old, G2P0, 27 weeks of gestation. Routine ultrasound examination suggested that fetus had multiple abnormalities, including fetal intracranial incomplete forebrain, cleft lip and palate, hypoplastic left heart syndrome (HLHS) and single umbilical artery. The couple chose to terminate the pregnancy and fetal muscle tissue was collected for CMA test.

#### Case 2

Thirty-five years old, G3P0, 9 weeks of gestation. The woman had three spontaneous miscarriages. Chorionic villus samples were collected for CMA test.

This study was approved by the Ethics Committee of Women’s Hospital, School of Medicine, Zhejiang University. Written informed consent was obtained from all of the participants.

### Chromosomal microarray

Genomic DNA samples were extracted with the GentraPuregene Kit (Qiagen, Germany). CMA was performed using the CytoScan™ 750 K array (Affymetrix, USA) according to the manufacturer’s instruction. The platform is composed of 550,000 non-polymorphic CNV probes and more than 200,000 SNP probes with an average resolution of 100 kb. All data were visualized and analyzed with the Chromosome Analysis Suite (ChAS) software (Affymetrix, USA). The reporting threshold of the copy number result was set at 1 Mb with marker count ≥50 for gains, 1 Mb with marker count ≥50 for losses. The analysis was based on the GRCh37/hg19 assembly.

### Multiplex Ligation-dependent Probe Amplification (MLPA)

MLPA was performed to confirm the CMA results by using subtelomeric MLPA kit P070-B2 (MRC-Holland, Amsterdam, The Netherlands). Three reference DNA samples from normal controls were used in each MLPA run. DNA was diluted into 35 ng/μl and 5 μl of DNA was used in each MLPA reaction. MLPA was performed according to the manufacturer’s recommendations and the PCR products were separated by capillary electrophoresis on ABI PRISM 3100 Genetic Analyzer (Applied Biosystems, CA, USA). Subsequent statistical and quantitative analyses were determined by Coffalyser Net software (MRC-Holland, Amsterdam, The Netherlands).

### Karyotyping

Peripheral blood samples from the parents were obtained and processed by standard chromosome procedures. GTG-banding analysis at 320–400 band resolution was performed with cultured cells [10].

### FISH

Peripheral blood samples of the Case 1 mother and her husband were analyzed by triple-FISH with the chromosome 6 subtelomeric probe (TEL6q, Spectrum orange, Vysis), the 17p subtelomeric probe (TEL17p, Spectrum Green, Vysis) and the chromosome 6 centromeric probe (CEP6, Spectrum Aqua, Vysis). For Case 2 couple, triple-FISH was performed with the chromosome 2 subtelomeric probe (TEL2q, Spectrum orange, Vysis), the 17p subtelomeric probe (TEL17p, Spectrum Green, Vysis) and the chromosome 17 centromeric probe (CEP17, Spectrum Aqua, Vysis). Probes used for FISH analysis of the other 5 cases were listed in Table 2. The slide hybridization and washes were performed according to standard FISH protocols [11]. Slides were counterstained with DAPI and analyzed under Zeiss ImagerA2 microscope (Zeiss, France). Image acquisition was subsequently performed using a CCD camera with Isis (FISH Imaging System, MetaSystems, Germany).

**Table 2:** Probes used in FISH analysis of 7 couples

Case	Probe^a^
Case 1	CEP6 Aqua; TEL17p SG; TEL6q SO
Case 2	CEP17 Aqua; TEL17p SG; TEL2q SO
Case 3	CEP16 Aqua; TEL16q SO; LSI13q14 SG
Case 11	TEL7p SG; CEP7 Aqua; TEL21q SO
Case 12	CEP15 Aqua; TEL15q SO; TEL20p SG
Case 13	TEL2q SG; TEL14q SO
Case 14	TEL3p SG; CEP9 Aqua; TEL9p SO

*SG* spectrum green, *SO* spectrum orange, *Aqua* spectrum aqua

^a^FISH test was performed on the metaphase of lymphocytes using telomeric probes

### Bioinformatics

To better understand the aberrations, we evaluated the duplicated and deleted regions with the information provided by the Online Mendelian Inheritance in Man database (OMIM, http://omim.org), the Database of Genomic Variants (DGV, http://dgv.tcag.ca/dgv/app/home), the DECIPHER Database (http://decipher.sanger.ac.uk) and the PubMed (http://www.ncbi.nlm.nih.gov/pubmed/).

## Results

A total of 1294 samples were tested by CMA during the study period, and 16 cases had partial trisomy for one chromosome and partial monosomy for the other chromosome. Subtelomeric MLPA confirmed the terminal deletions and duplications except that only the 3p deletion was confirmed in case 15 (Additional files 1 and 2: Figures S1 and S2). In 10/16 (62.5%) of these cases, CNVs were inherited from one carrier parent of balanced translocation (Cases 1 to 10), and 6/16 (37.5%) cases occurred de novo (Cases 11 to 16). Seven couples with normal karyotypes were performed FISH analysis for accurate diagnosis, and three of them were identified as submicroscopic balanced translocation carriers (Table 1). Following two cases showed representative cases of parental inheritance (recurrent miscarriage or congenital anomalies) which were initially given hints by CMA.

### Case 1

**Fig. 1:**
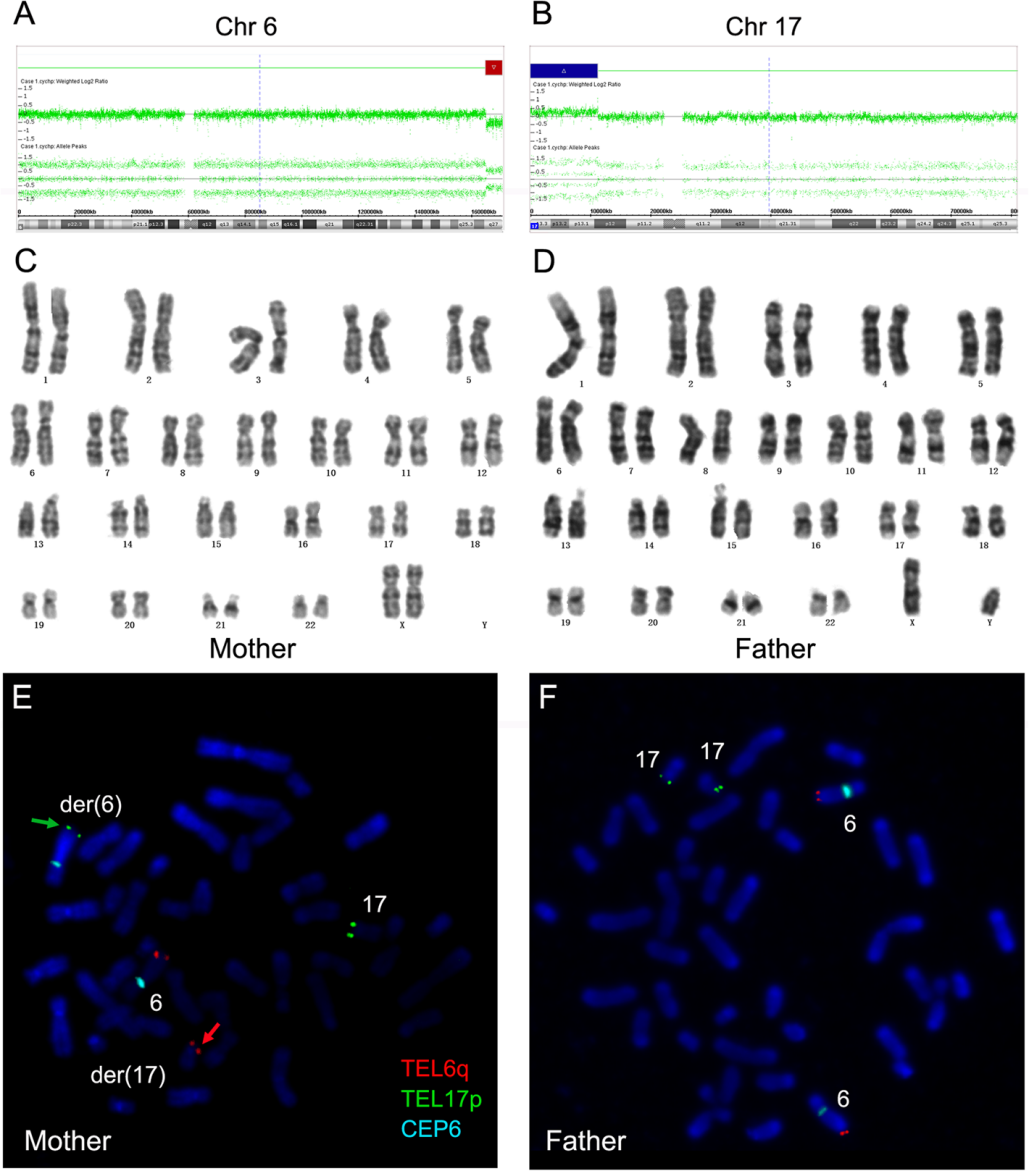
CMA analysis of POC from Case 1, and representative G-banding karyotypes and FISH analysis of parental peripheral blood. **a** The red bar indicates a 5.8 Mb deletion (6q27). The segment contained 22 OMIM genes; **b** The blue bar indicates the 17p13.3-p12 duplication, which was 11.2 Mb. The segment contained 171 OMIM genes; **c** and **d** G-banding karyotypes from metaphase peripheral blood lymphocytes of the Case 1 couples revealed normal karyotypes. **e** and **f** FISH results of the Case 1 couples with chromosome 6q subtelomeric probe (TEL6q SO, orange), chromosome 17p subtelomeric probe (TEL17p SG, green) and chromosome 6 centromeric probe (CEP6, aqua). Positive 6q signal indicated by the red arrow is found at the terminal short arm of the derivative chromosome 17, while positive 17p signal indicated by green arrow is found at the terminal of the derivative chromosome 6. The centromeres of chromosome 6 are shown by the aqua signals

The CMA analysis of POC from Case 1 showed a 5.8 Mb terminal deletion of 6q27 (chr6: 165,051,708–170,914,297) (Fig. 1a) and an 11.2 Mb terminal duplication of 17p13.3p12 (chr17: 525–11,208,838) (Fig. 1b). The deleted region contained 22 OMIM genes (Table 3), and the duplicated region included 171 OMIM genes (Table 3). Both of the parents are phenotypically healthy and have normal karyotype (Fig. 1c and d). Subtelomeric FISH revealed that the mother was a balanced translocation carrier, suggesting that the deleted chromosome 6q material observed in the CMA analysis was translocated with distal 17p segment (Fig. 1e and f).

**Table 3:** Summary of the CMA results of Cases 1and 2

Case	Type	Chr.	Region	OMIM genes
1	Loss	6	q27	*PDE10A, T, MPC1, RPS6KA2, RNASET2, FGFR1OP, CCR6, GPR31, UNC93A, TCP10, MLLT4, KIF25, DACT2, SMOC2, THBS2, PHF10, TCTE3, DLL1, FAM120B, PSMB1, TBP, PDCD2*
	Gain	17	p13.3p12	*DOC2B, RPH3AL, FAM57A, GEMIN4, RNMTL1, NXN, TIMM22, ABR, TUSC5, YWHAE, CRK, MYO1C, INPP5K, PITPNA, SLC43A2, SCARF1, RILP, PRPF8, MIR22, WDR81, SERPINF2, SERPINF1, RPA1, RTN4RL1, DPH1, OVCA2, MIR132, MIR212, HIC1, SMG6, SRR, TSR1, SGSM2, MNT, PAFAH1B1, OR1D2, ASPA, TRPV3, TRPV1, SHPK, CTNS, P2RX5, ITGAE, GSG2, CAMKK1, P2RX1, ATP2A3, ANKFY1, UBE2G1, SPNS3, SPNS2, MYBBP1A, GGT6, ALOX15, PELP1, ARRB2, MED11, CXCL16, ZMYND15, TM4SF5, PSMB6, PLD2, MINK1, CHRNE, GP1BA, SLC25A11, RNF167, PFN1, ENO3, SPAG7, CAMTA2, KIF1C, SLC52A1, ZFP3, USP6, SCIMP, RABEP1, NUP88, C1QBP, DHX33, DERL2, MIS12, NLRP1, AIPL1, PITPNM3, SLC13A5, XAF1, FBXO39, TEKT1, ALOX12, MIR195, BCL6B, CLEC10A, ASGR2, ASGR1, DLG4, ACADVL, DVL2, PHF23, GABARAP, CTDNEP1, ELP5, CLDN7, SLC2A4, YBX2, EIF5A, GPS2, ACAP1, KCTD11, TNK1, PLSCR3, NLGN2, SPEM1, TMEM102, FGF11, CHRNB1, ZBTB4, POLR2A, TNFSF12, TNFSF13, SENP3, EIF4A1, CD68, MPDU1, SOX15, FXR2, SHBG, SAT2, ATP1B2, TP53, WRAP53, EFNB3, DNAH2, KDM6B, CHD3, KCNAB3, TRAPPC1, CNTROB, GUCY2D, ALOX15B, ALOX12B, ALOXE3, HES7, PER1, VAMP2, AURKB, CTC1, PFAS, SLC25A35, RANGRF, ARHGEF15, ODF4, RPL26, NDEL1, MYH10, PIK3R6, PIK3R5, NTN1, STX8, WDR16, GLP2R, RCVRN, GAS7, MYH13, MYH8, MYH4, MYH1, MYH2, MYH3, SCO1, PIRT*
2	Gain	2	q37.1q37.3	*HTR2B, NCL, SNORD20, SNORD82, NMUR1, PTMA, PDE6D, NPPC, DIS3L2, ALPP, ALPPL2, ALPI, ECEL1, PRSS56, CHRND, CHRNG, TIGD1, EIF4E2, EFHD1, GIGYF2, KCNJ13, NGEF, NEU2, INPP5D, ATG16L1, SAG, DGKD, USP40, UGT1A8, UGT1A10, UGT1A9, UGT1A7, UGT1A6, UGT1A5, UGT1A4, UGT1A3, UGT1A1, HJURP, TRPM8, SPP2, ARL4C, SH3BP4, AGAP1, GBX2, CXCR7, COL6A3, MLPH, PRLH, RAB17, LRRFIP1, RAMP1, SCLY, HES6, PER2, TRAF3IP1, ASB1, TWIST2, HDAC4, NDUFA10, OTOS, GPC1, MIR149, RNPEPL1, CAPN10, GPR35, AQP12A, KIF1A, AGXT, PASK, PPP1R7, ANO7, HDLBP, SEPT2, STK25, BOK, THAP4, ATG4B, DTYMK, ING5, D2HGDH, GAL3ST2, NEU4*
	Loss	17	p13.3p12	*DOC2B, RPH3AL, FAM57A, GEMIN4, RNMTL1, NXN, TIMM22, ABR, TUSC5, YWHAE, CRK, MYO1C, INPP5K, PITPNA, SLC43A2, SCARF1, RILP, PRPF8, MIR22, WDR81, SERPINF2, SERPINF1, RPA1, RTN4RL1, DPH1, OVCA2, MIR132, MIR212, HIC1, SMG6, SRR, TSR1, SGSM2, MNT, PAFAH1B1, OR1D2, ASPA, TRPV3, TRPV1, SHPK, CTNS, P2RX5, ITGAE, GSG2, CAMKK1, P2RX1, ATP2A3, ANKFY1, UBE2G1, SPNS3, SPNS2, MYBBP1A, GGT6, ALOX15, PELP1, ARRB2, MED11, CXCL16, ZMYND15, TM4SF5, PSMB6, PLD2, MINK1, CHRNE, GP1BA, SLC25A11, RNF167, PFN1, ENO3, SPAG7, CAMTA2, KIF1C, SLC52A1, ZFP3, USP6, SCIMP, RABEP1, NUP88, C1QBP, DHX33, DERL2, MIS12, NLRP1, AIPL1, PITPNM3, SLC13A5, XAF1, FBXO39, TEKT1, ALOX12, MIR195, BCL6B, CLEC10A, ASGR2, ASGR1, DLG4, ACADVL, DVL2, PHF23, GABARAP, CTDNEP1, ELP5, CLDN7, SLC2A4, YBX2, EIF5A, GPS2, ACAP1, KCTD11, TNK1, PLSCR3, NLGN2, SPEM1, TMEM102, FGF11, CHRNB1, ZBTB4, POLR2A, TNFSF12, TNFSF13, SENP3, EIF4A1, CD68, MPDU1, SOX15, FXR2, SHBG, SAT2, ATP1B2, TP53, WRAP53, EFNB3, DNAH2, KDM6B, CHD3, KCNAB3, TRAPPC1, CNTROB, GUCY2D, ALOX15B, ALOX12B, ALOXE3, HES7, PER1, VAMP2, AURKB, CTC1, PFAS, SLC25A35, RANGRF, ARHGEF15, ODF4, RPL26, NDEL1, MYH10, PIK3R6, PIK3R5, NTN1, STX8, WDR16, GLP2R, RCVRN, GAS7, MYH13, MYH8, MYH4, MYH1, MYH2, MYH3, SCO1, PIRT, DNAH9, ZNF18, MAP2K4, MYOCD, ELAC2, HS3ST3A1*

No syndromes associated with the 5.8 Mb terminal deletion of 6q27 and 11.2 Mb terminal duplication of 17p13.3p12 were reported in the DECIPHER Database. Peddibhotla and his colleagues reported that seven unrelated patients with deletions involving chromosome 6q27 had structural brain abnormalities [12]. Neither of the two CNVs has been reported in the Database of Genomic Variants.

### Case 2

**Fig. 2:**
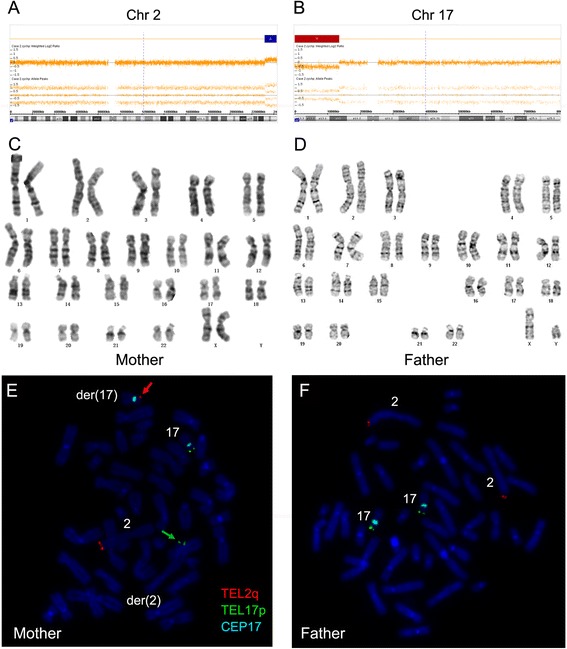
CMA analysis of POC from Case 2, and representative G-banding karyotypes and FISH analysis of parental peripheral blood. **a** The blue bar indicates the 2q37.1-q37.3 duplication, which was 10.8 Mb. The segment contained 82 OMIM genes; **b** The red bar indicates a 13.5 Mb deletion (17p13.3-p12). The segment contained 177 OMIM genes; **c** and **d** G-banding karyotypes from metaphase peripheral blood lymphocytesof the Case 2 couples revealed normal karyotypes; **e** and **f** FISH results of the Case 2 couples with chromosome 2q subtelomeric probe (TEL2q SO, orange), chromosome 17p subtelomeric probe (TEL17p SG, green) and chromosome 17 centromeric probe (CEP6, aqua). Positive 2q signal indicated by the red arrow is found at the terminal short arm of the derivative chromosome 17, while positive 17p signal indicated by green arrow is found at the terminal of the derivative chromosome 2. The centromeres of chromosome 17 are shown by the aqua signal

The CMA analysis of POC from Case 2 showed a 10.8 Mb terminal duplication of 2q37.1q37.3 (chr2: 231,919,970–242,782,258) (Fig. 2a) and a 13.5 Mb terminal deletion of 17p13.3p12 (chr17: 525–13,548,932) (Fig. 2b). The duplicated region covered 82 OMIM genes (Table 3), and the deleted region included 177 OMIM genes (Table 3). Both of the parents are phenotypically healthy and have normal karyotypes (Fig. 2c and d). Subtelomeric FISH showed that the duplicated chromosome 2q material observed in the CMA analysis was translocated with distal 17p segment (Fig. 2e and f). Therefore, the woman is the translocation carrier.

As shown by the DECIPHER Database, the 13.5 Mb terminal deletion of 17p13.3p12 covered the region of Miller-Dieker syndrome (MDS) which is characterized by lissencephaly (‘smooth brain’ or lack of normal gyri and sulci) with dysmorphic features. DECIPHER Database displayed that no syndromes were associated with 10.8 Mb terminal duplication of 2q37.1q37.3 region. Neither of the two CNVs has been reported in the Database of Genomic Variants.

## Discussion

In this study, POC samples from 16 cases had partial trisomy for one chromosome and partial monosomy for the other chromosome, and 10/16 (62.5%) of these cases, CNVs were inherited from one carrier parent of balanced translocation, diagnosed through karyotyping and FISH technology. Balanced translocation is the most common chromosomal abnormality with a frequency of 1 in 500 people [13, 14]. Although translocation carriers are usually phenotypically normal, they tend to produce a high percentage of unbalanced gametes and embryos due to chromosome imbalances during meiosis, resulting in infertility, recurrent miscarriage, and the birth of affected offspring [15, 16]. These various consequences depend mainly on the structural constitution of the translocated chromosome and the length of the chromosomal region involved. Despite there is a reasonable possibility of a healthy live birth following natural conception, these couples may choose to undergo preimplatation genetic diagnosis (PGD) treatment as an alternative to avoid consequences mentioned above. According to the documented literature, PGD technology is able to select embryos with normal or balanced translocation karyotype to transfer, avoiding fetal abnormalities caused by unbalanced translocation [17, 18]. In the present study, Case 4 had successfully given birth to healthy twin boys on Feb 2016 through PGD.

Routine G-banding karyotyping has a resolution ranging from 5 to 10 Mb [19]. However, when the chromosomal segmental imbalances were involved in atypical bands, or poor digestion and dyeing happened in the process of chromosome preparation, even CNVs larger than 10 Mb could be missed, such as Cases 1, 2 and 3. Case 3 is interesting because translocated segments 13q31.1q34 (34.4 Mb) and 16q12.1q24.3 (39 Mb) were both larger than 10 Mb, were of nearly equal length and having atypical bands. Our results finally showed that the husband of Case 3 mother is the carrier of translocation using FISH technology. It is worth mentioning that such cases would easily be mistakenly diagnosed as de novo genomic imbalance if FISH analysis had not been taken into account. On the other hand, high resolution chromosome analysis has the advantage of recognizing smaller structural abnormalities over conventional karyotyping. FISH analysis was utilized to compensate for the misdiagnosis caused by low resolution in conventional karyotyping.

With parental studies, namely karyotyping and/or FISH technologies, we are able to determine the inheritance of the structural rearrangements. In this retrospective study, the origins of unbalanced translocation derivative were uncovered in all the 16 cases. Ten cases were caused by parental balanced translocation while the remaining 6 chromosomal abnormalities were occurred de novo. Although de novo mutations were found in cases 11–16, we could not exclude the possibility of germline mosaicism in these cases. Subtelomere FISH testing in addition to karyotyping are also useful in diagnosing the causes of miscarriage for couples with multiple pregnancy losses. However, these two technologies are unable to disclose all the reasons of miscarriage due to complicated and diversified causes such as infection and immune system responses.

Taken together, CMA testing on POC samples plays an important role in identifying chromosome translocation, especially for couples who carries balanced translocation but with normal karyotypes (Cases 1, 2 and 3). Accurate diagnosis of parental chromosome translocation can well be achieved through only FISH, but FISH analysis would not be performed normally unless CMA of POC showed CNVs. Parents who were diagnosed as a balanced translocation carrier could consider PGD, or have a natural conception in future pregnancy, and conventional karyotyping together with CMA should be applied in the prenatal diagnosis.

## Conclusion

CMA testing is becoming increasingly important as a diagnostic tool for detecting chromosome abnormalities on POC samples. This study underscores the importance of CMA testing on POC samples together with parental karyotyping and FISH analysis to allow for a more refined and precise diagnosis on whom carries a balanced translocation.

## Additional files

Additional file 1: Figure S1. MLPA analysis with the kit P070-B2 confirmed the terminal deletions and duplications of Case 1 to 9. Red asterisks indicate the deleted regions, and blue asterisks indicate the duplicated regions. (TIFF 13493 kb)

Additional file 2: Figure S2. MLPA analysis with the kit P070-B2 confirmed the terminal deletions and duplications of Case 10 to 16. Red asterisks indicate the deleted regions, and blue asterisks indicate the duplicated regions. (TIFF 11476 kb)
