# Supplementary figures and images for: A feasible diagnostic approach for the translocation carrier from the indication of products of conception
Source: Mol Cytogenet. 2018 Jan 30;11:12. doi: 10.1186/s13039-018-0362-8 (PMC5791184; doi:10.1186/s13039-018-0362-8)

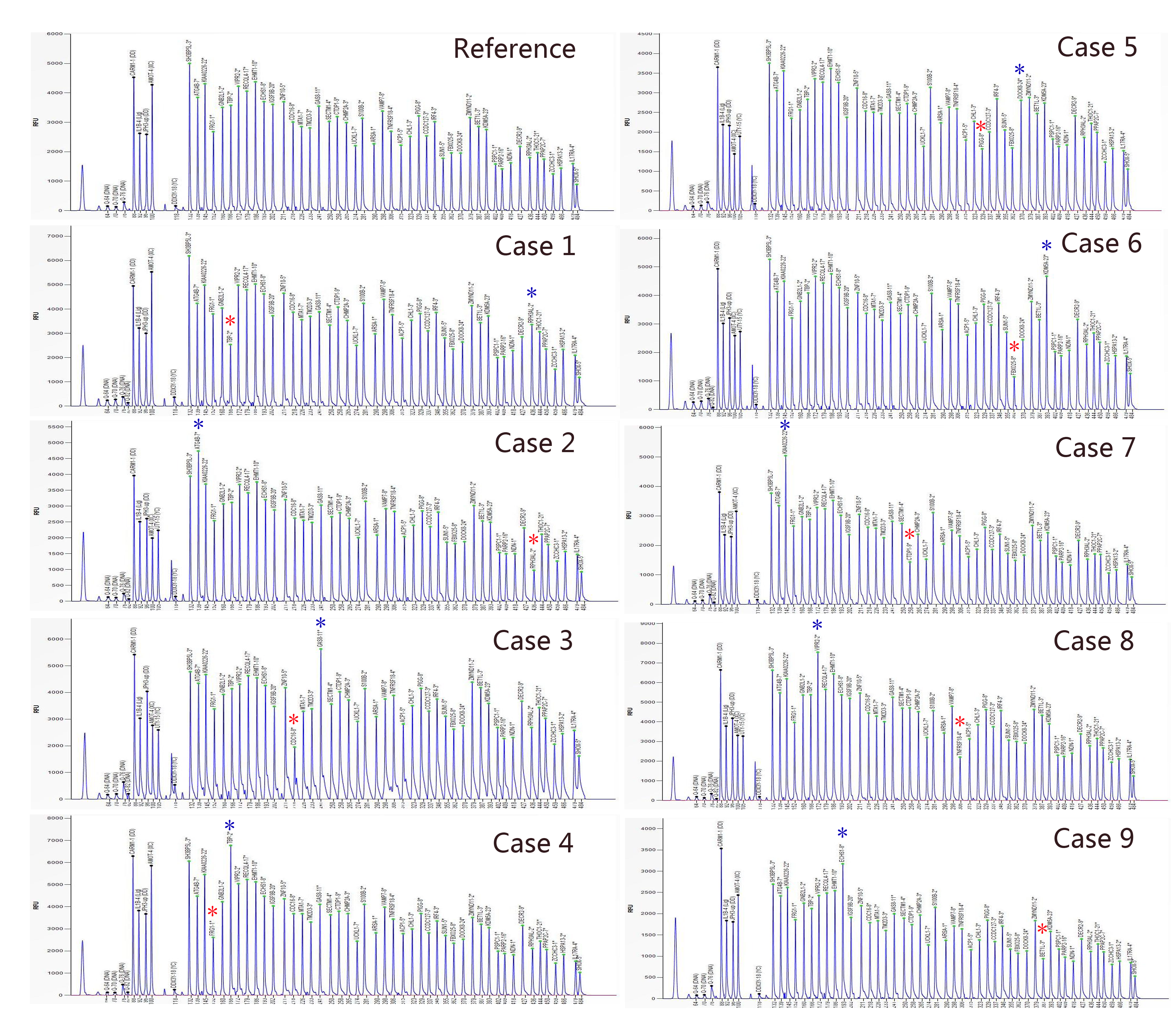

Supplement: Supplementary file 1 — MLPA analysis with the kit P070-B2 confirmed the terminal deletions and duplications of Case 1 to 9. Red asterisks indicate the deleted regions, and blue asterisks indicate the duplicated regions. (TIFF 13493 kb) [file 13039_2018_362_MOESM1_ESM.tif]

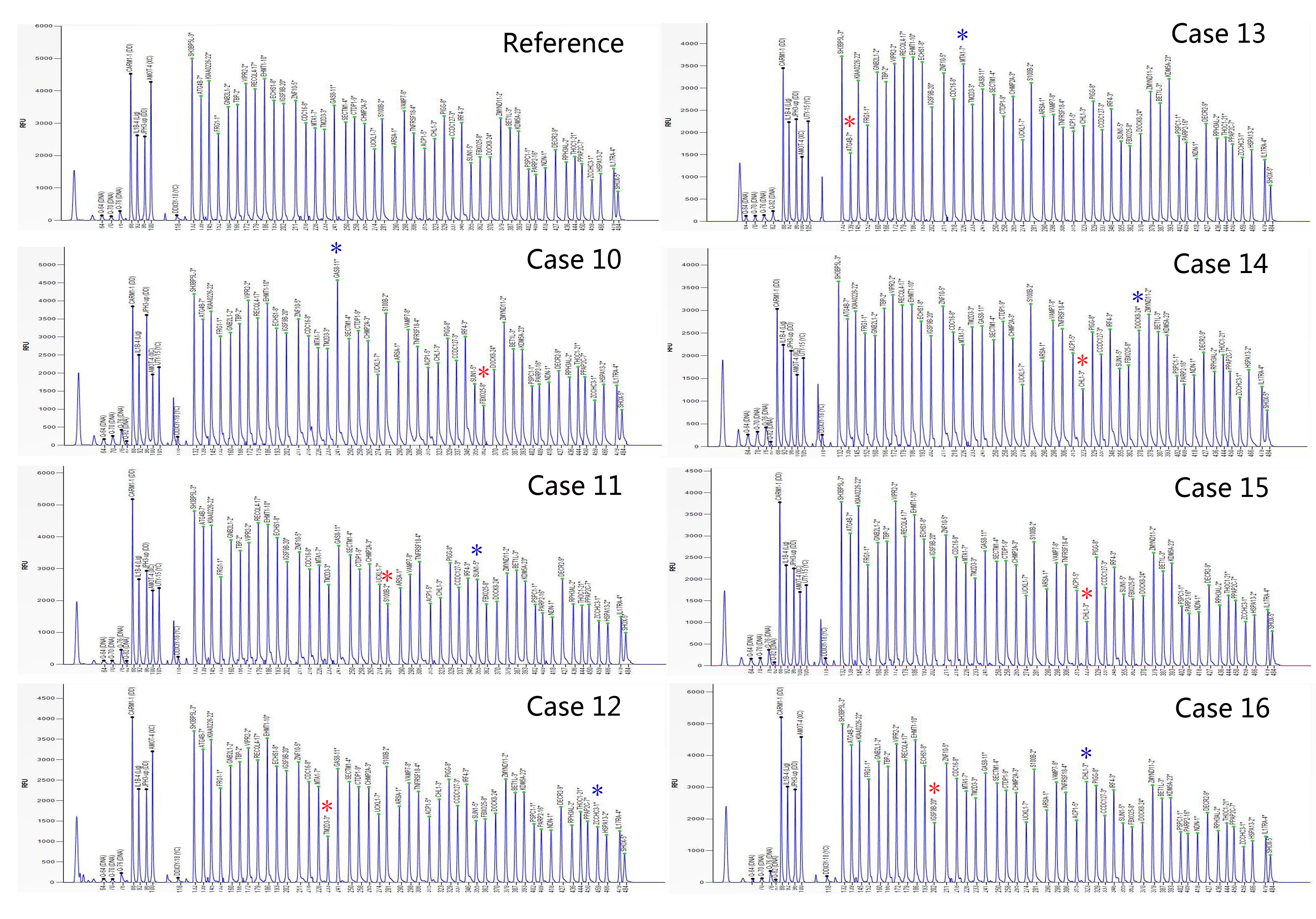

Supplement: Supplementary file 2 — MLPA analysis with the kit P070-B2 confirmed the terminal deletions and duplications of Case 10 to 16. Red asterisks indicate the deleted regions, and blue asterisks indicate the duplicated regions. (TIFF 11476 kb) [file 13039_2018_362_MOESM2_ESM.tif]
